# Contrast Agents of Magnetic Resonance Imaging and Future Perspective

**DOI:** 10.3390/nano13132003

**Published:** 2023-07-04

**Authors:** Jie Lv, Shubham Roy, Miao Xie, Xiulan Yang, Bing Guo

**Affiliations:** 1 School of Computer Science and Engineering, Yulin Normal University, Yulin 537000, China; 2 Shenzhen Key Laboratory of Flexible Printed Electronics Technology, School of Science, Harbin Institute of Technology, Shenzhen 518055, China; shubham.roy111@yahoo.in; 3 Shenzhen Key Laboratory of Advanced Functional Carbon Materials Research and Comprehensive Application, School of Science, Harbin Institute of Technology, Shenzhen 518055, China

**Keywords:** MRI, machine learning, deep learning, ChatGPT, contrast agents

## Abstract

In recent times, magnetic resonance imaging (MRI) has emerged as a highly promising modality for diagnosing severe diseases. Its exceptional spatiotemporal resolution and ease of use have established it as an indispensable clinical diagnostic tool. Nevertheless, there are instances where MRI encounters challenges related to low contrast, necessitating the use of contrast agents (CAs). Significant efforts have been made by scientists to enhance the precision of observing diseased body parts by leveraging the synergistic potential of MRI in conjunction with other imaging techniques and thereby modifying the CAs. In this work, our focus is on elucidating the rational designing approach of CAs and optimizing their compatibility for multimodal imaging and other intelligent applications. Additionally, we emphasize the importance of incorporating various artificial intelligence tools, such as machine learning and deep learning, to explore the future prospects of disease diagnosis using MRI. We also address the limitations associated with these techniques and propose reasonable remedies, with the aim of advancing MRI as a cutting-edge diagnostic tool for the future.

## 1. Introduction

The translation of science and technology from the bench to the bedside leads us to several futuristic technologies in the field of biomedical engineering. One of the most appropriate examples of this is surely the magnetic resonance imaging (MRI) [1]. On the one hand, MRI reveals any internal inflammation, tumor, images of blood vessels, tissue perfusion, soft tissues, and characterization of pathological sites, etc., to easily diagnose diseases [2,3]. On the other hand, it provides superior noninvasiveness, minimal toxicity (in the case of contrast agents), and maintains bio- and cytocompatibility if compared to other imaging modalities such as X-rays and computed tomography (CT), etc. [4]. In reality, MRI uses strong magnetic fields to align protons in any biological sample and exploits their longitudinal (T1) or transverse (T2) relaxations to produce images. Thus, it is necessary to polarize the water molecules as much as possible to obtain a good MR image with high contrast. However, in the case of the brain or any interior organs, the contrast becomes dull.

Herein, scientists have developed magnetic materials to enhance the contrast of the MR signals. MRCAs can be broadly classified into two distinct groups, namely T1-based agents and T2-based agents [5,6]. Contrast agents belonging to both of these groups are equally important in imaging; however, their mechanistic approaches are slightly different from each other. While T1-based MRCAs use the longitudinal signal for relaxation, the T2-weighted MRCAs use a transverse signal. From the diagnostic point of view, both of these classes show enhanced MR contrast upon intravenous injection. Among various MR contrast agents (MRCAs), gadolinium (Gd^3+^)-based agents have shown significant development in past decades [7]. Owing to its large spin quantum state, Gd^3+^ has seven unpaired electrons (S = 7/2), making it favorable for magnetic applications. Notably, almost 40% of Gd-based MRCAs can be used for full-body MR imaging, and 60% of them can be successfully used in neurological MRI [8].

However, it has been recently observed that Gd-based MRCAs can cause nephrogenic systemic fibrosis (NSF) and could be harmful in different parts of the human body [4]. It may also be dangerous to use these contrast agents on patients having acute kidney or liver diseases [9]. In fact, it is also not advised to use Gd-agents in renal imaging. Hence, there is a pressing need to overcome such barriers. Herein, the advent of alternative contrast agents become crucial in the past few years. Apart from lanthanides (Gd^3+^, Dy^3+^, Eu^3+^), transition metal ions can also be fruitful in this domain owing to their large spin polarization [10]. Herein, Fe^3+^ and Mn^2+^ have become favorable choices as T1 MRCAs. Mn^2+^ contrast agents have shorter Mn-H_water_ bond lengths with a favorable high number of unpaired electrons in their valent states (S = 5/2) [11]. Moreover, it can efficiently cross the blood–brain barrier (BBB) through cerebrospinal fluid, cerebral capillaries, or the olfactory nerve, which makes Mn-complexes effective for neuroimaging. Several groups have recently developed some fascinating Mn^2+^ MRCAs for imaging different parts of the human body. Most of these studies validated the fact that Mn-based complexes could aid MRI in the near future, and they could possibly overcome the toxicity of the Gd^3+^ [4].

**Figure 1:**
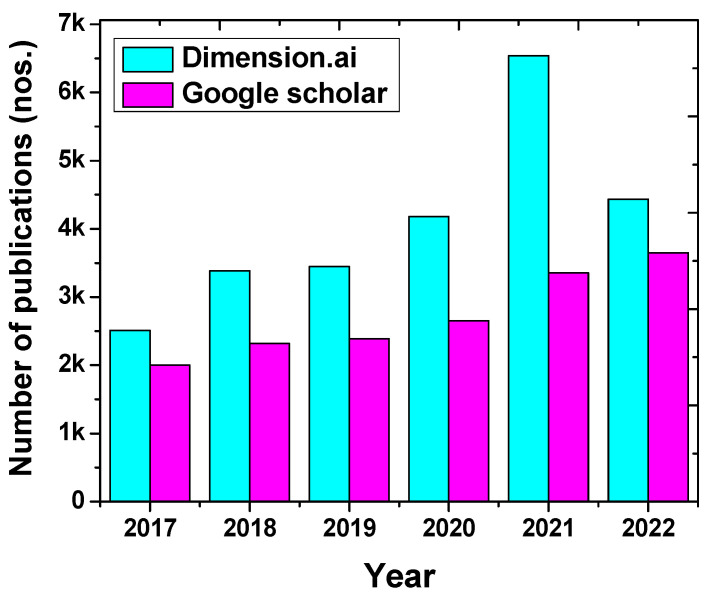
The number of papers published in the last six years (2017–2022) on the Mn-based MRI CAs.

The sudden emergence of Mn^2+^ MRI CAs has predominantly taken over the domain of contrast agents and thereby gives rise to numerous review articles in recent times. Notably, more than 3000 articles each year from the past five years have been published globally, which certainly signifies the demand for more research in this field (Figure 1). On the one hand, the review articles demonstrate the overall progress and the need for Mn-based contrast agents in MRI [4,12,13], while Zhang and his team suggested Mn-based MOFs for MRI imaging [11]. Here, they summed up the recent developments of MOF-mediated theranostic platforms, which are capable of performing chemotherapy as well.

On the other hand, scientists are taking a step forward towards the efficient targeting of the cells/organs by the CAs. Notably, such targeting strategies can enhance the contrast by accumulating inside the specified organs/cells. However, most of these targeting techniques are still on the bench owing to their stability and other limitations. Herein, several image-processing tools have come up, showing enormous potential in the domain of molecular imaging. Strategies like artificial intelligence (AI), more precisely, data science, and machine learning have elevated the quality of the images to the peak [14,15]. In this review, the rational design of the MRI CAs has been explored. Special emphasis has been given to their targeting strategies and their limitations. Afterward, numerous image processing techniques have been discussed that can improve image quality significantly. Several recent examples have also been demonstrated to discuss the very recent progress of digital molecular imaging. We hope this work will substantiate the understanding of MRI CAs and the image processing tools, which will surely make this modality a futuristic tool to diagnose and treat severe diseases.

## 2. MRI Contrast Agents: Principle, Disease Diagnosis, and Recent Progress

Nowadays, almost one-third of the MRIs have been performed under the guidance of contrast agents [16,17]. Clinically Gd^3+^ has become the most dominant contrast agent until now. However, recent research reveals the need for alternative contrast agents like Fe^3+^ and Mn^2+^ for better visibility and bioavailability [18,19]. In this section, a discussion has been made on such contrast agents and their rational designing approaches. In addition, a comparative discussion has also been made between Mn-based MRCAs and Gd-based MRCAs, including their diagnostic efficiency, disease identification, and underlying basic photophysics.

**Figure 2:**
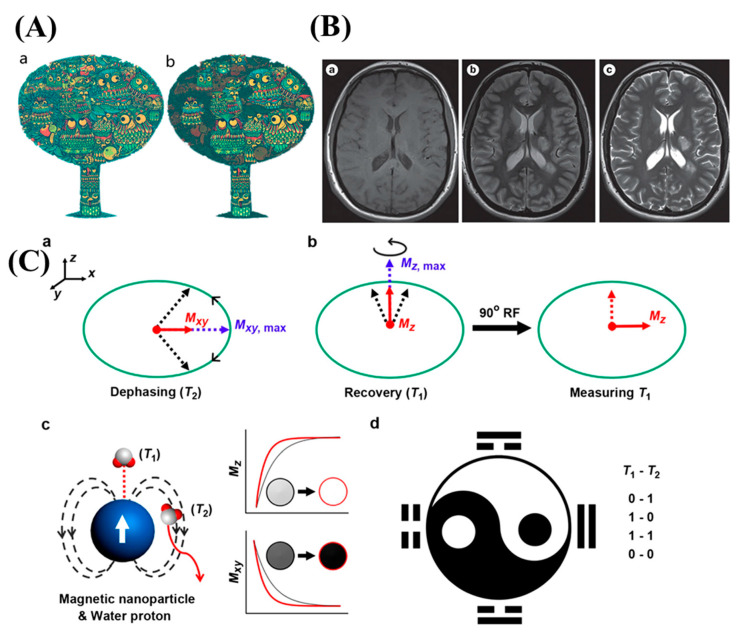
(**A**) Nature has a fondness for mimicry, and in a similar vein, radiologists have a preference for accentuating lesions when it comes to medical diagnostics. While a regular MRI (**a**) may reveal the subject of examination to some extent, it often lacks clear boundaries. However, employing an external contrast agent (**b**) can aid in emphasizing particular pathologies, thereby assisting in their identification. Copyright (2017) with permission from the American Chemical Society [25]. (**B**) (**a**) Before administering the contrast agent, (**b**) axial T1-weighted and (**c**) dual-echo T2-weighted sequences are obtained. Copyright (2015) with permission from Springer-Nature [26]. (**C**) (**a**) T2 relaxation is associated with the loss of magnetization in the xy plane (*M_xy_*) following a 90° radiofrequency (RF) pulse. The maximum *M_xy_* immediately after nuclear magnetic resonance is denoted as *M_xy,max_*. (**b**) T1 relaxation refers to the recovery of magnetization in the z direction (*M_z_*) from zero to the maximum value (*M_z,max_*) while the nuclei are spinning. To measure M_z_, another RF pulse is applied to flip the magnetization from the z direction to the xy plane. The T2 decay effect can attenuate the T1 effect due to this additional pulse. (**c**) The T1 relaxation enhancement is directly related to the coordination with a magnetic nanoparticle, while the T2 relaxation enhancement is associated with the diffusion around the nanoparticle. This results in brighter contrast in T1 imaging and darker contrast in T2 imaging when it comes to water protons. (**d**) T1-T2 dual-modal imaging operates on a logic of different states: OFF-ON (0-1), ON-OFF (1-0), ON-ON (1-1), and OFF-OFF (0-0). These states offer new possibilities for demonstrating multiple parameter features in magnetic resonance imaging (MRI). Copyright (2017) with permission from the American Chemical Society [27].

In reality, the protons from the water or fat content of the body align with the magnetic field given and thereby relaxes in order to promote a signal. However, in normal MRI, such magnetic polarization depends on various factors, and the external field cannot align all protons simultaneously. Hence, contrast agents are in high demand in the case of rigorous and spontaneous MR imaging. It is observed that materials with many unpaired electrons in their valance shell could create high spin-polarization and, thereby, superior magnetic moment (Figure 2) [20].

From the biophysical and biochemical perspective, these contrast agents can be divided into four distinct groups, namely superparamagnetic, paramagnetic, chemical exchanges and transfer-based, and direct detection based. Among them, para- and superparamagnetic contrast agents have occupied the domain for the past few decades [21,22]. Most clinical contrast agents fall into these two categories, such as manganese, gadolinium, iron, etc. Gadolinium-based contrast agents have been used most compared to the other MRCAs. The first clinically approved contrast agent was gadolinium diethylenetriamine pentetic acid [Gd(DTPA) (H_2_O)]^2−^, which was approved back in 1988 [23,24].

T1 relaxation occurs as the external magnetic field approaches when spins experience energy loss as a result of interactions with the surrounding environment. Through interactions between water molecules and the metal ions in the contrast agent’s core, the contrast agent speeds up the loss of energy. T1 contrast agents function best when their rotational motion corresponds to the Larmor frequency (42.58 MHz T^−1^ for hydrogen protons) when energy transfer is most effective [28]. The hydrogen protons lose energy and regain their original magnetic moment more quickly as a result of interaction with the contrast agent, which decreases the water molecules’ rapid tumbling motion to a frequency near the Larmor frequency. The magnetization in the transverse plane is net reduced, as seen by the T2 relaxation [29]. A little magnetic moment is produced as a result of the initial net alignment with the external magnetic field, becoming a net phase coherence of their precessions in the transverse plane. The precessional motion of these spins toward decarbonization is promoted by all processes leading to T1 relaxation, whereas T2 relaxation is influenced by different mechanisms. Contrast agents also alter T2 by causing localized inhomogeneities in the magnetic field. Regions where the applied longitudinal field and Larmor frequency differ are produced by the induced fields. Since spins are out of phase due to the realignment caused by the precession of hydrogen protons, the net transverse magnetization decreases. In general, Equation (1) can be used to express the relaxation time (*T_i_*) of water protons, where Ti0 is the intrinsic relaxation time of the tissues and *T_i_* CA is the contribution from the contrast agent. An agent’s longitudinal (*r_1_*) and transverse (*r_2_*) relaxivities can be used to quantify its contrast ability. According to Equation (2), the relaxivity values express the degree to which a contrast agent can increase the hydrogen nucleus relaxation rate constant Ri normalized to the agent concentration [30].

(1) $$\frac{1}{T_{i}}=\frac{1}{T_{i0}}+\frac{1}{T_{i}^{CA}}\cdots i=1,\;2$$

(2) $$R_{i}^{CA}=\frac{1}{T_{i}^{CA}}=r_{i}\left[CA\right]\cdots i=1,\;2$$

Contrast agents having significant limits of detection (LOD) with smaller dosages are more desirable. In the case of MRI, a very small amount of contrast agent (in μM level) is needed. However, the administrated dose may be fairly high in most cases, which raises concerns about safety and secondary toxicity. This is why very few MRCAs have been approved by the US Food and Drug Administration (FDA) [31]. Thus, it is crucial to design contrast agents, which not only can perform better but also have minimal or no side effects.

**Figure 3:**
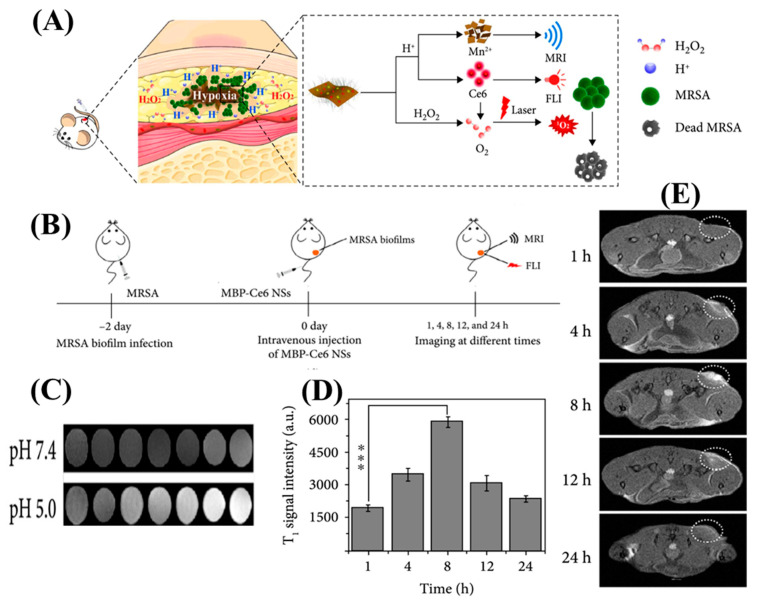
(**A**) (A) This study focuses on preparing MnO_2_-BAS/PEG-Ce6 nanosheets (MBP-Ce6 NSs) and their application in responsive FL/MR imaging and enhanced antimicrobial photodynamic therapy (aPDT) for bacterial biofilm infections. (**B**) A schematic illustration depicts the process of detecting MRSA biofilms using MBP-Ce6 NSs through intravenous injection. (**C**) T1-weighted MR images of MBP-Ce6 NSs solutions in PBS are captured, demonstrating different concentrations and pH values. (**D**) The intensity of the T1-weighted MR signal in MRSA biofilm-infected tissues is measured at various time points after the injection of MBP-Ce6 NSs. *** *p* < 0.001 (two-tailed Student’s *t*-test). (**E**) Cross-sectional T1-weighted MR images are shown, and the corresponding intensity of the T1-weighted MR signal in MRSA biofilm-infected tissues is analyzed at different times post-injection. Copyright (2020) with permission from Science [33].

MRI is an important tool to diagnose any inconsistencies present in the interior section of the human body. It can successfully detect deep-seated infections, vasculature diseases, inflammatory diseases, neurodegenerative disorders, and tumors. Thus, this technology is useful in diagnosing any disease rapidly and quantitively. On the other hand, MRI contrast agents (CAs) provide several other features in addition to disease detection, such as they can act as theranostic modalities and can act as image-guiding surgical and therapeutic systems. For instance, Li et al. worked on a self-assembling peptide (FFYEGK) with a Gd-complex and vancomycin for detecting and ablating *S. aureus* infection in vivo. The Gd-complex was used here as the MRI imaging probe conjugated with the antibacterial vancomycin. The peptide in the complex played an important role in increasing the longitudinal relaxivity rate (*r_1_*) after self-assembly owing to the π–π stacking. The high relaxivity value of the theranostic agent may be attributed to its unique nano-aggregated structural features, which would limit molecular rotation and extend the time that Gd-chelates would spend tumbling [32]. In addition, Xiu and his team demonstrated a MnO_2_ NP-derived system that has an extraordinary ability to image bacterial biofilm (Figure 3). In this study, the authors have successfully increased the T1 relaxation time of the Mn^2+^-based MRI agent by bacterial microenvironment-responsive MRI. After 8 h of subcutaneous injection, the contrast agent attains the highest relaxation and simultaneously reduces after 24 h, indicating the excretion of the sample from the kidneys [33].

MRI imaging has also been exploited in tumor diagnosis. Zhao and his team successfully detected tumors in the liver by using MRI. It is challenging to perform MRI in the liver due to the toxicity of the Gd-based CAs. Herein, the authors did not utilize any CAs for MRI imaging tumors [34]. Not only the liver, but MRI can also efficiently diagnose brain tumors by passing through the blood–brain barrier (BBB).

**Figure 4:**
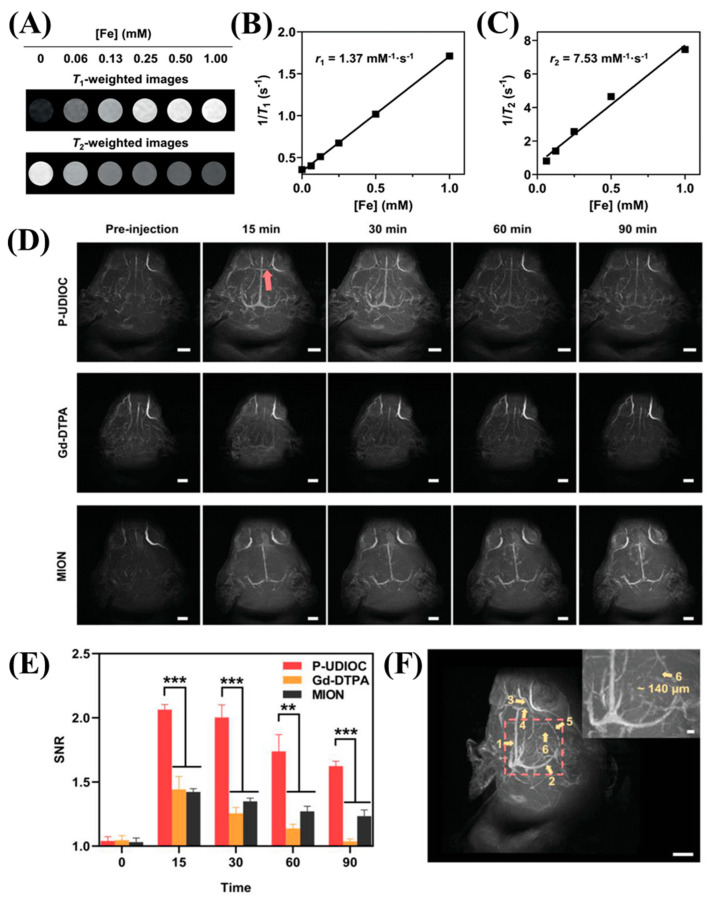
(**A**) T1-weighted and T2-weighted magnetic resonance (MR) images of P-UDIOC are acquired at 7 T. (**B**) The T1 relaxivity, indicated by the slope, and (**C**) the T2 relaxivity, also indicated by the slope, of P-UDIOC are measured at 7 T. (**D**) Maximum intensity projection images of the rat brain using P-UDIOC, Gd-DTPA, and MION as contrast agents are displayed. The scale bar represents 5 mm. The red arrow represents the location of the contrast. (**E**) Vascular imaging performance is quantitatively analyzed by calculating signal-to-noise ratio (SNR) values of the inferior cerebral vein, before and after intravenous injection of P-UDIOC, Gd-DTPA, and MION. (Sample size: *n* = 3). **, *** describes the statistical error margin. (**F**) A three-dimensional (3D) volume image of the rat brain captured at 15 min post-injection of P-UDIOC demonstrates high spatial resolution of the vasculature and the circle depicts the contrasting area. The scale bar represents 5 mm. Copyright (2021) with permission from Wiley [35].

The current progress of MRI in medical imaging enables it to image the vascular anatomy and its functions quite significantly. On the one hand, low-field MRI (<3 T) suffers from significant spatiotemporal resolution and offers low-contrast images. On the other hand, there are almost no CAs for ultra-high field MRI (UHF-MRI) (>7 T), which makes it challenging to image vasculature diseases with ease. Very recently, scientists have been working on iron-based dual T1-T2 CAs in order to mediate this gap. For instance, Wang et al. demonstrated a T1-T2 dual-mode iron oxide nanoparticle-based contrast agent (UDIOC) for the detection of vasculature diseases under UHF-MRI. The ultrasmall core size of these NPs makes them useful in this domain with a promising pharmacokinetic profile. This CA can be exploited for images as small as 140 μm diameters, extending the detection limit of the 7 T MR angiography (Figure 4) [35]. In a similar manner, several Gd and transition metal-based CAs have emerged as promising agents for MRI-mediated vasculature imaging [36,37]. However, more research is needed to keep the toxicity low and maintain feasible excretion levels in order to get translated into clinics.

**Figure 5:**
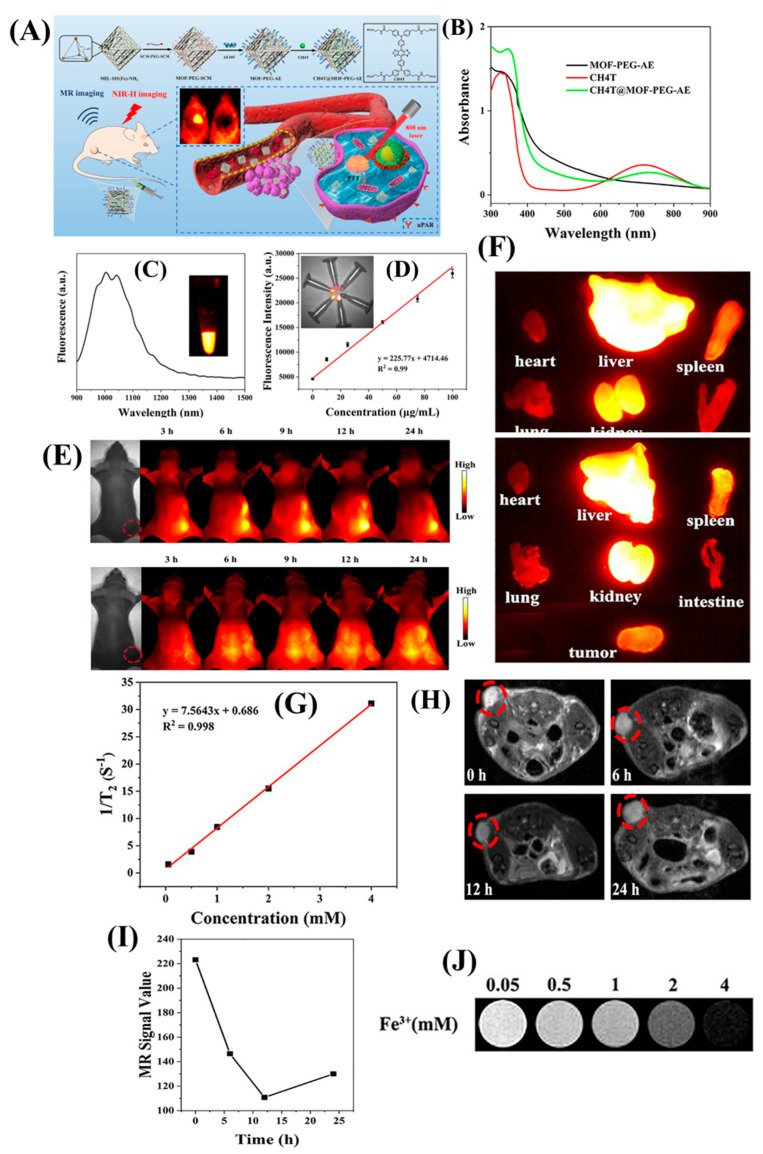
(**A**) This figure depicts the process of fabricating the CH4T@MOF-PEG-AE theranostic nanoplatform and its applications in imaging-guided photothermal cancer therapy and surgery of orthotopic glioblastoma. (**B**) The absorbance spectra of MOF-PEG-AE, CH4T, and CH4T@MOF-PEG-AE aqueous solutions are recorded. (**C**) NIR-II fluorescence emission spectra of CH4T@MOF-PEG-AE in water are measured using an 808 nm laser for excitation. (**D**) A linear fit is performed to correlate the fluorescence intensity with the concentration of CH4T@MOF-PEG-AE. (**E**) CH4T@MOF-PEG-AE or CH4T@MOF-PEG-SCM (tumors highlighted by red circles) are observed. (**F**) Ex vivo NIR-II fluorescence images of major organs and tumors are captured after treatment with CH4T@MOF-PEG-AE or CH4T@MOF-PEG-SCM. (**G**) T2-weighted MR images and T2 relaxation curves of CH4T@MOF-PEG-AE are acquired for different Fe concentrations (mM). (**H**) T2-weighted MR images and (**I**) corresponding MR signal values of the xenograft GBM model are obtained before and after treatment with CH4T@MOF-PEG-AE (tumors highlighted by red circles). (**J**) MR signals of the samples. Copyright (2021) with permission from Elsevier [40].

MRI has also been explored in predicting neurodegenerative disorders and various inflammatory diseases [38,39]. The spatiotemporal response and contrast of the images are fairly high in such cases making this diagnostic modality an eminent choice in clinics. The MRI CAs are generally conjugated with NIR-II emitters for effective in vivo NIR-II imaging-guided MRI. For example, Li et al. diagnosed glioblastoma using NIR-II fluorophore (CH4T) loaded Fe-based metal-organic framework (MOF) nanoparticles and modified with tumor-targeting AE105 peptide [40]. The nanoplatform can invade through the BBB layer, and the peptide efficiently targets the over-expressed urokinase Plasminogen Activator Receptor (uPAR) in glioblastoma cells (Figure 5). NIR-II facilitated high detection sensitivity and spatiotemporal resolution, essential to delineate the cancer cells accurately, along with the advantages of high penetration depth of magnetic resonance imaging.

As evident from various studies, doping Mn into CuS nanoparticles can enhance T1/T2 MRI contrast and exhibit a high absorption in the NIR region, which is suitable for PA imaging [41]. CuS-incorporated Mn nanoparticles and surface coated with PEG or bovine serum albumin (BSA) can sufficiently provide enhancement in both the PA and MRI signals in targeted cells [42] For MRI/PAI, Yang et al. fabricated a nanoscale metal-organic particles (NMOPs) using Mn and a NIR dye IR825, and coated with PEG functionalized polydopamine (PDA) shell to offer strong contrast in T1-weighted MR imaging. Under an 808 m laser, the biodistribution and tumor therapy was visualized. At 3.0 T, relaxivity *r1* of 7.48 mM^−1^ s^−1^ was observed, and the nanoprobe got completely eliminated within 60 days of administration [43].

## 3. Disease Targeting Using the MRI CAs

**Figure 6:**
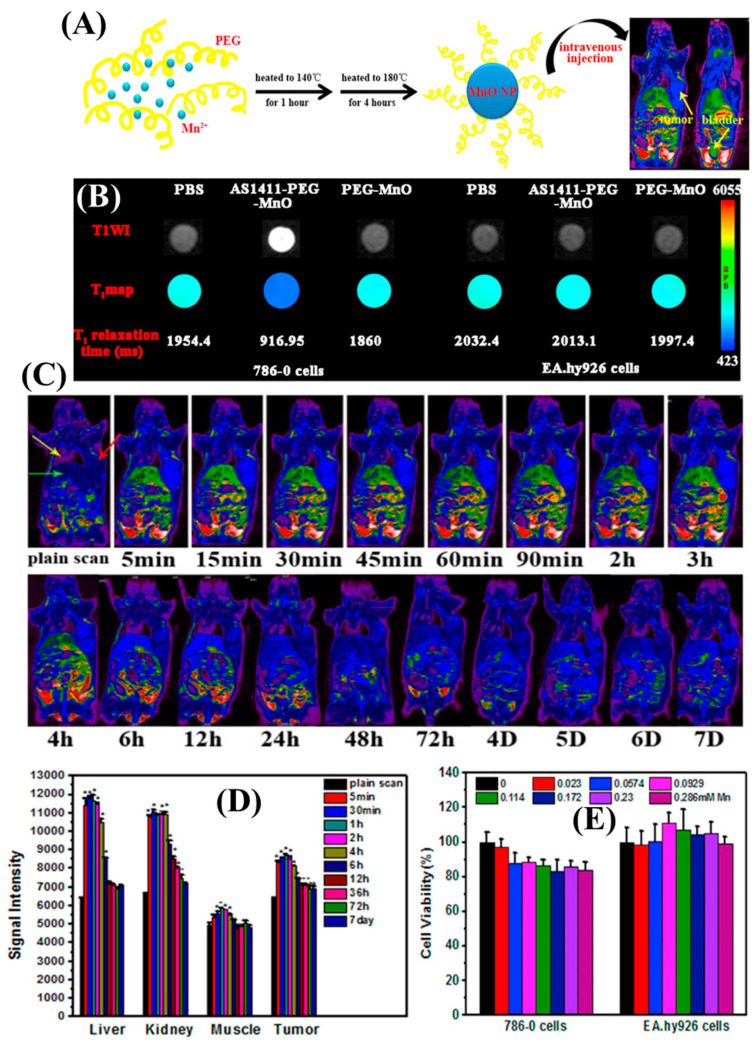
(**A**) The process of one-pot preparation of hydrophilic PEG-MnO nanoparticles for in vivo magnetic resonance imaging (MRI) of renal carcinoma is illustrated. (**B**) T1-weighted MR images, T1 maps, and corresponding T1 relaxation times are shown for 786-0 renal carcinoma cells treated with PBS, AS1411-PEG-MnO nanoprobe, or PEG-MnO nanoparticles. Control cells (EA.hy926) were treated similarly. (**C**) Pseudo-color MR T1 images are presented for mice with renal carcinoma tumors before and after the injection of AS1411-PEG-MnO nanoprobe. Tumors, heart, and liver are indicated by red, yellow, and green arrows, respectively. (**D**) Signal intensities of various organs (liver, kidney, muscle, and tumor) are measured before (plain scan) and after (5 min, 30 min, 60 min, 2 h, 4 h, 6 h, 12 h, 24 h, 36 h, 72 h, 7D) intravenous injection of AS1411-PEG-MnO nanoprobe. The data is collected from three samples. * *p* < 0.001 compared to plain scan. (**E**) Cell viability of 786-0 renal carcinoma cells and normal human umbilical vein endothelial cells (EA.hy926) is assessed using the MTT assay after exposure to different concentrations of PEG-MnO nanoparticles. Copyright (2018) with permission from Elsevier [44].

In the current scientific world, to enhance the targeting ability and for better contrasting effects, certain manganese-coated nanoparticles are modified with various aptamers. For example, Li and his colleagues adopted a facile one-pot approach to developing T1 MRI nano-contrast agent using hydrophilic MnO nanoparticles with PEG-bis (carboxymethyl) ether 600 as the solvent, and covalently cross-linked amine group functionalized AS1411 aptamer for detection of brain disease [44]. High T1 relaxivity (12.942 mM^−1^ s^−1^) and a low r2/r1 ratio (4.66) were observed at 3.0 T, along with high stability in the glutathione environment. Target-specific MRI using AS1411-PEG-MnO nanoprobe was evaluated against renal carcinoma (Figure 6). The contrast of different organs (heart, kidney, muscle, liver) of the renal carcinoma-bearing mice showed increased contrast that reached a maximum after 45 min post-injection, specifically at the tumor region. The signals disappeared completely at 24 h post-injection, suggesting complete clearance of the nanoprobe, mainly through the kidneys and bladder.

For the purpose of the target-specific release of cargo, increased colloidal stability, dispersibility, solubility, and biocompatibility, and most importantly, for enhancing T1-weighted MRI, the Mn nanoparticles are often modified with various chemical moieties or polymers (PVP polyglycerol adipate, or polyethyleneimine) [30]. PEG-phospholipid is one of the most popular stabilizing ligands for MnO nanoparticles [45]. Since the phospholipid micelles have a tight hydrophobic lining, it provides a protective barrier to the Mn nanoparticles, thus reducing the efficiency of water exchange which lower the *r*1 relaxivity. On the contrary, hydrophilic coating material will positively influence the efficiency of water exchange and influence the T1 relaxation time. Moreover, the binding of Mn nanoparticles to biological receptors can be regulated when the terminal reactive groups adjoin with PEG moieties. For instance, Wang et al. modified MnO nanoparticles with L-cysteine and PEG for T1-weighted MRI. While PEG enhanced the stability, L-cysteine improved blood circulation time and reduced cellular uptake by macrophages [46].

In addition, MRI-guided theranostics have become a popular choice among clinicians. The chemodynamic therapy (CDT) is based on the initiation of oxidative stress due to an acidic tumor microenvironment (overexpressed GSH and H_2_O_2_) that results in Haber-Weiss or Fenton reaction and generation of reactive species like •OH, ^1^O_2_, or •O_2_^−^, which ultimately causes tumor suppression by necrosis or apoptosis of the tumor cells [47,48].

The presence of Mn^2+^ can initiate •OH radical formation from the endogenous H_2_O_2_ via Fenton or Fenton-like reactions [49]. Apart from chemodynamic therapy, MRI-guided other therapies have initiated a lot of research. Zhang et al. provided a novel idea for reversing MDR tumor chemotherapy using MnO_2_/DOX-loaded albumin nanoparticles (BMDN) [50]. The MDR reversal through interaction with albumin receptors which are overexpressed on cancer cells includes reduced drug efflux, achieved on-demand drug release, enhanced cellular uptake, and decreased hypoxic tumor microenvironment. The acidic microenvironment promotes the release of Mn^2+^, which enhances the contrasting effect, thus causing good in vitro and in vivo T1-weighted imaging. On the other hand, MRI-guided surgery has drawn a lot of importance recently. Salem et al. demonstrated a Laser interstitial thermal treatment (LITT), which uses an MRI to guide the procedure and uses heat from a laser to target and destroy certain tissues or lesions. For individuals who cannot endure surgery, LITT offers a nonsurgical alternative that is thought to be less intrusive than open surgery [51].

Thus, it is evident that microenvironment-based targeting and/or other tumor-targeting strategies of these new-generation MRI CAs can be fruitful in ensuring better imaging quality and specificity. Moreover, these targeting strategies can be crucial to localize the CAs to minimize their dose and, thereby, secondary toxicities. These targeting modalities also pave the path toward the new age theranostics regimes for better visualization and therapy.

## 4. Future of MRI Imaging: AI and Image Processing Techniques

Machine learning and deep learning are two main pillars of artificial intelligence. On the one hand, where machine learning utilizes a set of given data to construct a logical resection of the problem, deep learning uses brain-like neural networks to perform the specific job. These two terminologies offer similar results; however, their underlying principles are quite different. Recently, deep learning and machine learning (ML) techniques have been broadly used in MRI-mediated diagnosis [52,53]. These modern techniques can enhance image quality or may be useful in diagnosing the spread of the disease.

### 4.1. Machine Learning (ML) in MRI

ML shares a significant contribution from the past few years in the domain of molecular imaging analysis. The disease detection and estimation of their spread have been successfully conducted by several groups meanwhile. For instance, Beatriu Reig and the team demonstrated several machine-learning models to identify breast cancers. They state how improvements in lesion detection, lesion classification, radiogenomics, and neoadjuvant chemotherapy response prediction, the field of ML in breast MRI is quickly developing. Both supervised and unsupervised ML algorithms need more research because they have not yet been put to use in clinical settings. This work shows a comparative outlook between different ML classifiers and their diagnosis abilities of breast cancer [54].

**Figure 7:**
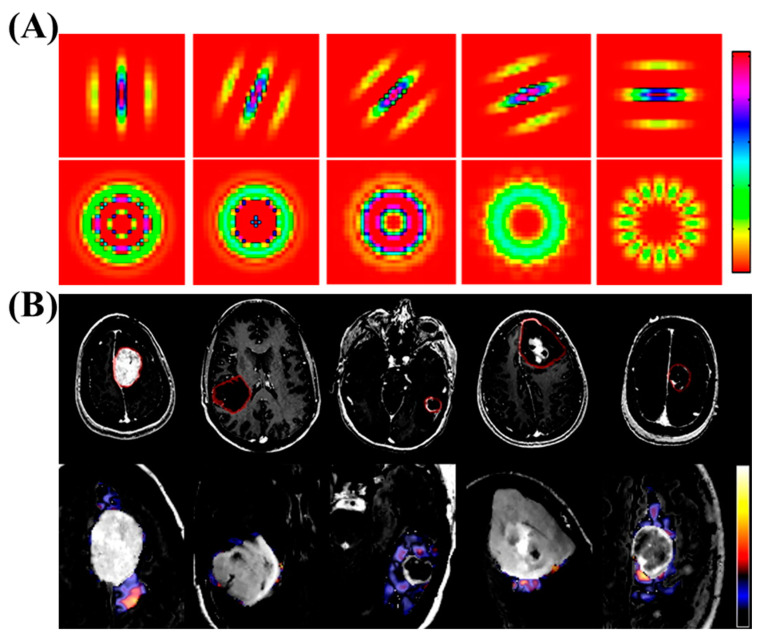
(**A**) The figure displays examples of filters utilized for extracting texture features. The first row demonstrates Gabor filters with the same frequency but different orientations, while the second row showcases rotation-invariant filters. (**B**) MR images of various brain tumor types are shown, along with an example of texture images extracted from the edematous area. The tumors depicted include meningioma, glioma grade II, grade III, grade IV, and metastasis. In the first row, the tumoral region of interest is displayed using the T1ce image. In the second row, a zoomed-in FLAIR image of the tumor region is overlaid with one of the textural patterns (λ = 8). It is important to note that the presented pattern, shown as a voxelwise texture, is for illustrative purposes and does not reflect our actual calculations. The average texture values, calculated prior to the fast Fourier transform, are also indicated. Copyright (2009) with permission from Wiley [55].

Similarly, ML has successfully been used in diagnosing brain tumors. Zacharki et al. used the textures and sizes of the tumors in MRI images to diagnose whether it is a primary glioma or metastases. This study subsequently grades the glioma by using a pattern classification method. The proposed plan involves a series of steps, such as identifying the area of interest, extracting features, selecting relevant features, and classifying the brain tumors. The extracted features will include the shape and intensity characteristics of the tumor, along with texture features that are rotation invariant. To narrow down the features, a support vector machine with recursive feature elimination will be used. The approach was tested on a group of 102 brain tumors that were diagnosed with different types of cancer, including glioblastoma, meningiomas, and metastasis. Using the binary support vector machine classification method with leave-one-out cross-validation, they achieved an accuracy of 85% in distinguishing metastasis from gliomas, and 88% accuracy in discriminating high-grade tumors from low-grade tumors. The sensitivity for differentiating metastasis was 87%, and the specificity was 79%. The sensitivity and specificity for distinguishing high-grade tumors from low-grade tumors were 85% and 96%, respectively (Figure 7) [55].

In addition, the prognosis of another severe neurodegenerative disorder, namely Alzheimer’s disease, can also be made by using ML methods. Moradi and his team suggested a new way of detecting the conversion from mild cognitive impairment (MCI) to Alzheimer’s disease (AD) was created using magnetic resonance imaging (MRI) scans. An aggregate biomarker was developed, which is called the MRI biomarker, using a combination of semi-supervised and supervised learning algorithms. Some unique characteristics of this technique include: (1) a semi-supervised approach was used to construct the MRI biomarker, (2) features were selected from MRI data of AD subjects and normal controls without using MCI subject data, (3) the effects of aging were removed from the MRI scans, and (4) the MRI biomarker was combined with other data such as age and cognitive measures of the MCI subjects to create the aggregate biomarker. Data from the Alzheimer’s Disease Neuroimaging Initiative (ADNI) database was used to prove the effectiveness of the technique. It was found that the MRI biomarker was able to distinguish between progressive and stable MCI patients with a 10-fold cross-validation area under the receiver operating characteristic curve (AUC) of 0.7661 [56].

**Figure 8:**
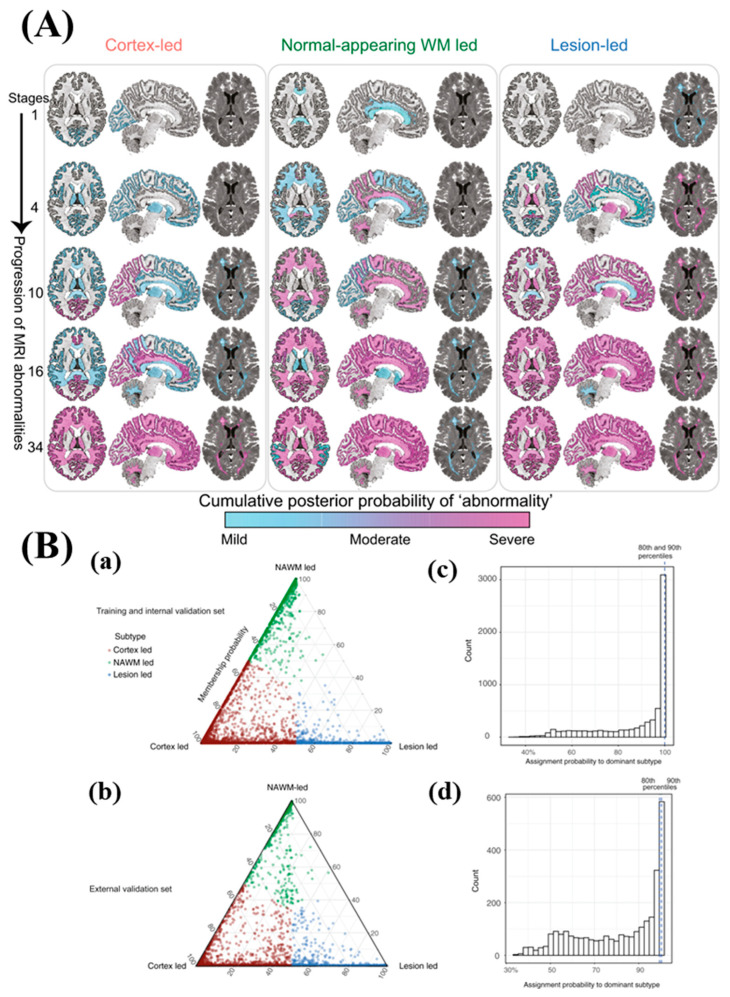
(**A**) The figure illustrates the progression of MRI abnormalities in each of the three MRI-based subtypes. The color gradient ranges from blue to pink, representing the probability or degree of abnormality (mild, moderate, or severe, which can be approximated as 1, 2, and 3 sigmas). (**B**) (**a**) The MRI-based subtypes are depicted in the training and internal validation dataset, and (**b**) in the validation dataset. The assignability of the disease subtype, or membership probability, is indicated by the distance from each vertex of the triangle. Each vertex represents the point at which membership to a specific subtype is at its maximum (100%). Subjects are assigned to one subtype based on their maximum probability, shown in red, green, and blue. (**c**,**d**) The 80th and 90th percentiles for the probability of assignment to the dominant subtype were 99.98% and 99.99% (indistinguishable in the figure) in the training dataset. In the validation dataset, these percentiles were 99.45% and 99.97%, respectively. Copyright (2021) with permission from Springer-Nature [57].

ML has also been successfully exploited in diagnosing Multiple sclerosis (MS), which is subdivided into four main phenotype groups. Groups with similar features can be identified using multidimensional data through machine learning. In this study, unsupervised machine learning is applied to brain MRI scans acquired in previously published studies in order to classify MS subtypes based on pathological features. MRI-based subtypes are defined using a training dataset from 6322 MS patients, and validation is conducted using an independent cohort of 3068 patients. MS subtypes are defined as cortex-led, normal-appearing white matter-led, and lesion-led based on the earliest abnormalities observed. The lesion-led subtype is associated with the highest risk of confirmed disability progression (CDP) and the highest relapse rate. Positive treatment response in selected clinical trials is observed in individuals with the lesion-led MS subtype (Figure 8). It is suggested by our findings that MRI-based subtypes can predict MS disability progression and treatment response, and they may be utilized to define groups of patients in interventional trials [57].

Apart from these works, scientists have worked on other diseases, such as identifications of prostate cancer, cardiovascular diseases, etc. [58,59]. Owing to its activity in different disease detection, ML could be an outstanding tool to diagnose deep-seated diseases in the near future if properly improvised. This method needs more research and simultaneous attention by scientists and clinicians.

### 4.2. Deep Learning (DL) in MRI

With the maturation of deep learning architectures, previous state-of-the-art classical machine learning algorithms are gradually being outperformed (Table 1). DL has been used in several sectors of disease diagnosis and image processing. Recently, an overview of current deep learning-based segmentation approaches for quantitative brain MRI is aimed to be provided in the review article of Akkus et al. Firstly, the deep learning architectures currently utilized for the segmentation of anatomical brain structures and brain lesions are reviewed. Subsequently, the performance, speed, and properties of deep learning approaches are summarized and discussed. Lastly, the current state is critically assessed, and potential future developments and trends are identified. This review provides various dimensions in the domain of MRI imaging of brain tumors. According to their study, it is found that computer-aided techniques have faced a great challenge in the analysis of brain images, primarily due to the complex anatomy and variability of brain appearance, non-standardized MR scales resulting from variations in imaging protocols, imperfections in image acquisition, and the presence of pathology. As a result, there is a requirement for more generic techniques, such as deep learning, which can effectively handle these variabilities. However, despite a significant breakthrough, the potential of deep learning is limited in the context of medical imaging datasets. This limitation arises from the relatively small size of these datasets, which hampers the ability of the methods to fully demonstrate their power when compared to their performance on large-scale datasets (e.g., millions of images) like ImageNet [60].

In addition, Liu and his team highlighted different deep learning architectures, such as artificial neural networks, deep feedforward networks, stacked autoencoders, deep belief networks, etc., to demonstrate the feasibility of the DL in decoding the MRI. They also depicted various applications of deep learning in this domain, such as image detection, image registration, image segmentation, and image classifications [61].

Like machine learning, DL has also been exploited in various disease diagnoses. In their study, a deep neural network was trained by Pinaya et al. to diagnose patients with SCZ (schizophrenia) from healthy controls using MRI images. The network combined a deep belief network with a softmax layer to extract high-level latent features. The training process involved two steps: (1) pre-training the network using a deep belief network, and (2) performing supervised fine-tuning using a softmax layer. The pre-trained network was utilized to identify high-level latent features from the MRI images, while the softmax layer refined the pre-trained network through supervised fine-tuning and facilitated the final classification process [62]. Similarly, a deep learning approach was proposed by Suk et al. to diagnose Alzheimer’s disease (AD) and its prodromal stage, Mild Cognitive Impairment (MCI). The method involved extracting high-level latent and shared features from two imaging modalities: MRI images and Positron Emission Tomography (PET) images. Discriminative patches between classes were obtained using a statistical significance test. Subsequently, a multimodal deep Boltzmann machine was constructed to discover high-level latent and shared features from these paired patches. Within the multimodal deep Boltzmann machine, a Gaussian Restricted Boltzmann Machine was trained to transform the paired patches into binary vectors. These binary vectors served as inputs to the multimodal deep Boltzmann machine. Following the identification of high-level latent and shared features through the paired patches and trained multimodal deep Boltzmann machine, an image-level classifier was developed for the final classification. The construction of the classifier involved three main steps: (1) learning of the patch-level classifier, (2) construction of mega-patches, and (3) ensemble learning [63]. These DL techniques are very crucial for new-age MR image processing. However, DL needs rigorous research before clinical translation for accuracy.

### 4.3. OpenAI (ChatGPT and GPT-4) and MRI

The current status of ChatGPT, its uses, ethical implications, and distinctive features are explored in this section, highlighting the power of this AI chatbot. The significance of artificial intelligence in modern technology cannot be overstated, as it operates in the background, simulating human intelligence and assisting us in various tasks. The continuous evolution and development of AI have brought about revolutionary advancements in this field. ChatGPT exemplifies the remarkable progress achieved in artificial intelligence, introducing numerous groundbreaking innovations. This section delves into the nature of ChatGPT, examines its availability as a free service, explores its applications, and addresses the ethical concerns associated with its usage. The fundamental understanding of ChatGPT necessitates a brief overview of its underlying principles. Functioning as an exceptionally advanced chatbot, OpenAI, an American artificial intelligence research company, serves as the mastermind behind the development of ChatGPT.

**Figure 9:**
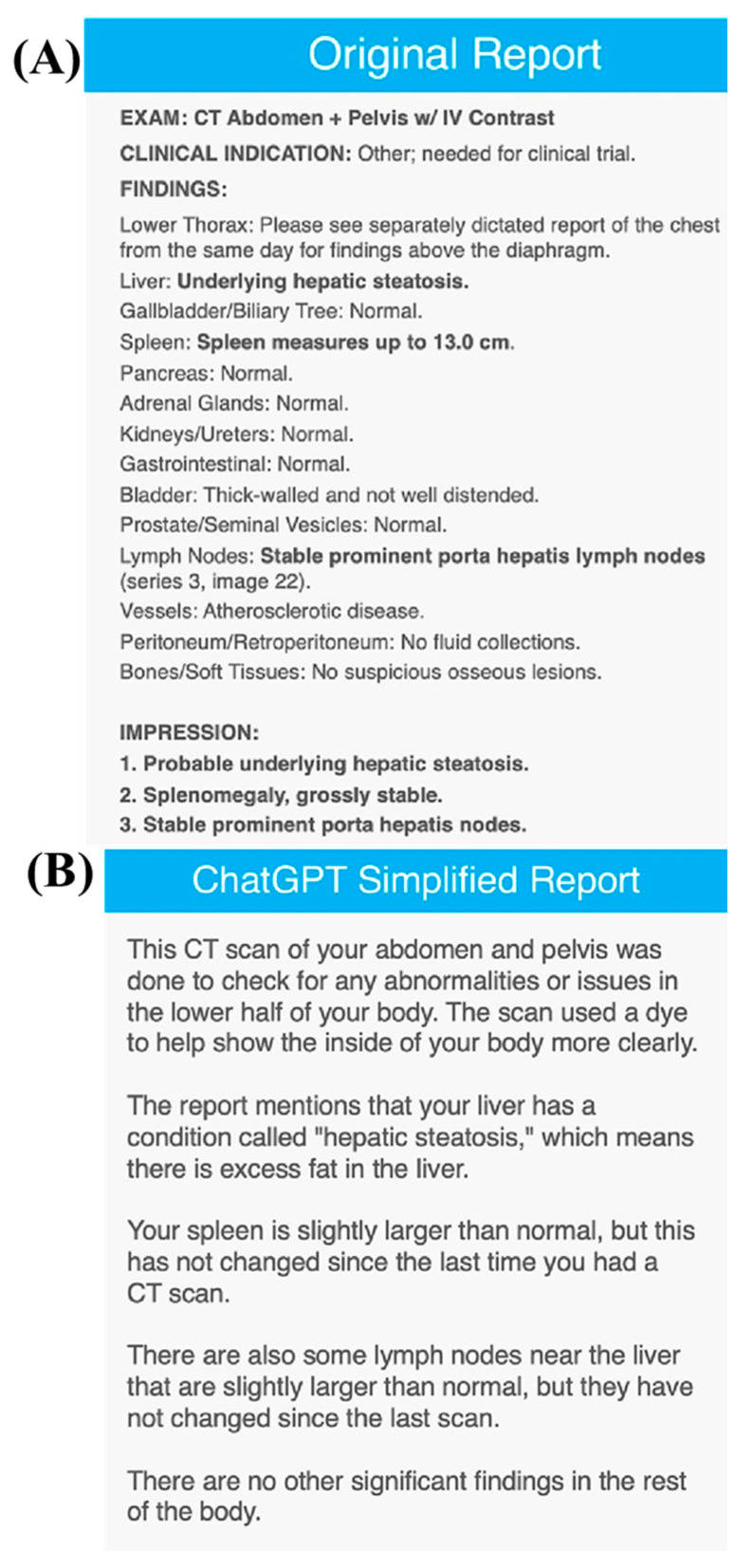
The simplified reports generated by ChatGPT for MRI scans. Copyright (2023) with permission from Elsevier [64].

**Figure 10:**
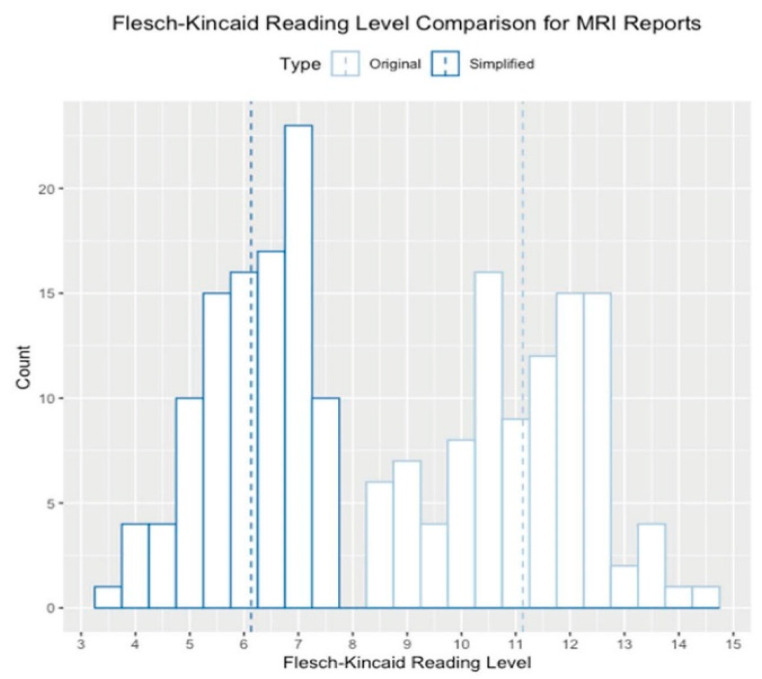
The histogram displays the reading level distribution of both the original reports and Copyright (2023) with permission from Elsevier [64].

Notably, ChatGPT and similar AI tools have also been utilized in understanding the MRI and other molecular imaging results. Li et al., in their very recent paper, exploited ChatGPT to summarize and decode radiology reports of patients. It was found that a total of 400 radiology reports, including 100 radiographs (XR), 100 ultrasound (US), 100 CT, and 100 MRI reports, were randomly sampled from our institution’s database dated between 2022 and 2023. These reports were subjected to processing by ChatGPT using the prompt “Explain this radiology report to a patient in layman’s terms in the second person: <Report Text>. “ For each report and ChatGPT output, the mean report length, Flesch reading ease score (FRES), and Flesch-Kincaid reading level (FKRL) were calculated. The determination of significance was conducted using *t*-tests. The mean report length was found to be 164 ± 117 words, with FRES averaging 38.0 ± 11.8 and FKRL averaging 10.4 ± 1.9. A significant difference was observed in FKRL, as it was higher for CT and MRI in comparison to US and XR. A FKRL value below 8.5 was only observed in 60 out of 400 cases (15%). The mean length of the simplified ChatGPT output was 103 ± 36 words, with FRES averaging 83.5 ± 5.6 and FKRL averaging 5.8 ± 1.1. Notably, a mean reduction of 61 words (*p* < 0.01) was observed, accompanied by an increase in FRES by 45.5 (*p* < 0.01) and a decrease in FKRL by 4.6 (*p* < 0.01). Importantly, all simplified outputs had an FKRL value below 8.5 (Figure 9 and Figure 10) [64].

**Figure 11:**
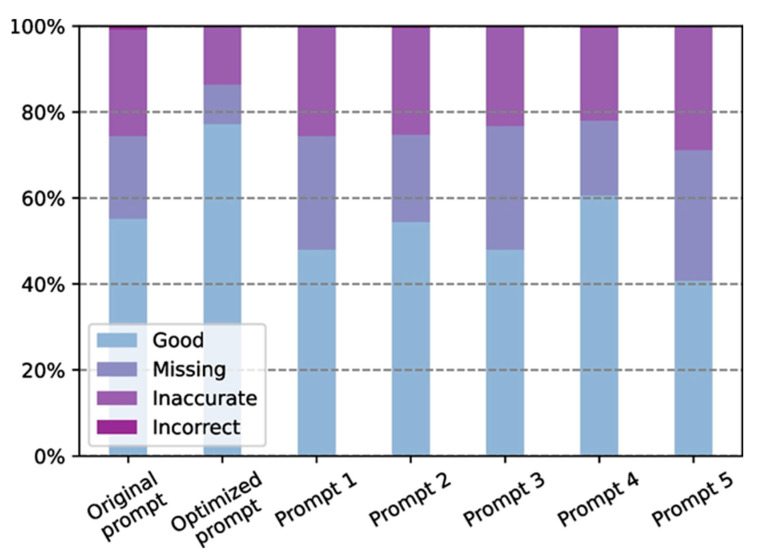
The impact of various prompts on the translation performance of ChatGPT was examined and evaluated. Copyright (2023) with permission from Springer-Nature [65].

However, some groups have opposite thoughts and evidence against such AI tools in decoding imaging reports. For instance, Lyu and his team demonstrated how simple clinical keywords can affect the results in ChatGPT. They indicated the feasibility of utilizing ChatGPT in experiments aimed at translating radiology reports into plain language for patients and healthcare providers, with the goal of improving healthcare education. For this study, radiology reports were collected from 62 low-dose chest computed tomography lung cancer screening scans and 76 brain magnetic resonance imaging metastases screening scans conducted. According to the assessment performed by radiologists, successful translation of radiology reports into plain language was achieved using ChatGPT, with an average score of 4.27 in a five-point system. The evaluation indicated a minor information gap of 0.08 and a minimal presence of misinformation at 0.07. In terms of the suggestions provided by ChatGPT, they were generally relevant, including recommendations for continued follow-up with doctors and close monitoring of symptoms. Out of a total of 138 cases, specific suggestions based on the findings in the report were offered by ChatGPT in approximately 37% of the cases. However, ChatGPT exhibited occasional randomness in its responses, occasionally oversimplifying or neglecting certain information (Figure 11). This issue can be addressed by employing a more detailed prompt [65].

In our opinion, more research is needed on such AI tools before doing any serious events, such as medical or any other report analysis and decoding. Scientists and clinicians must follow a rational approach and multilayered rigorous quality control checking before translating them into the clinics.

## 5. Summary and Outlook

This work initially reports the fundamental understanding of MRI contrast agents, their rational design, and limitations, which needs to be resolved soon. Thereafter, the authors provided the targeting strategies of MRI contrast agents to obtain improved image quality and better contrast. Such designing approach and targeting strategies would definitely give a better understanding to the new researchers and clinicians in this domain. The examples and case studies in these sections are recently published and demand more focus on the mainstream MRI CAs. Herein, some theranostic MRI CAs and multimodal MRI probes have been discussed to rationalize their acceptance in the near future in clinics.

In addition, special emphasis has been given to various image-processing modalities. In the recent past, rigorous attention has been paid to machine learning and deep learning-based AI techniques, which have been covered with numerous case studies and examples. Their limitations have also been illustrated in order to substantiate their clinical translation probabilities in the near future. Furthermore, very recent AI tools such as ChatGPT and their advantages and limitations regarding the MRI result analysis have been depicted, which contemplates the fact that there is a long way to go before making them translatable into the clinics.

However, such image-processing AI techniques embedded with conventional MRI and rationally designed biocompatible CAs are the future of MRI and other molecular imaging modalities. Surely, these modifications could pave the path towards a futuristic imaging regime, which could be translated easily from the bench to the bedside.

## Figures and Tables

**Table 1:** Different AI tools to perform DL.

Sl. No.	Program Name	Website (Accessed on 18 June 2023)
1	PyTorch	https://pytorch.org/
2	CNTK	https://www.microsoft.com/en-us/cognitive-toolkit/
3	TensorFlow	https://www.tensorflow.org/
4	Theano	http://www.deeplearning.net/software/theano/
5	Keras	https://keras.io/
6	Torch	http://torch.ch/
7	Caffe	https://caffe.berkeleyvision.org/
8	Chainer	https://chainer.org/
9	DeepLearning4j	https://deeplearning4j.org/
10	FastAI	https://www.fast.ai/

## Data Availability

No data has been used in this work.
